# Travis County Medical Society; Proceedings

**Published:** 1890-02

**Authors:** B. F. Church

**Affiliations:** Secretary


### TRAVIS COUNTY MEDICAL SOCIETY.

Regular meeting for the report of cases, December 12, 1889. Dr. T. J. Tyner, president, in the chair.

Dr. Morris reported a case of puerperal eclampsia followed by septic infection. Ten grains of antipyrine, with five of pulv. doveri was sufficient, in each instance, to control the exarcebation of fever, the temperature remaining down from four to eight hours, and accompanied by profuse perspiration.

Dr. Weller, a case of unusually prompt conception after delivery. The child—apparently at full term—was born just nine months and twenty days after the birth of one fully developed and at full term.

Dr. Bennett called attention to the danger of producing epididymitis in some cases of stricture of the urethra, by the introduction of sounds. Saw it caused at three different times, in the same subject, by their use.

Dr. Denton related the history of a case of pedunculated submucous fibroid tumor of the uterus. Hemorrhage more or less profuse, had existed at intervals for five or six months. The tumor was grasped with forceps, drawn through the os, and excised. Complete recovery soon followed.

Dr. Tyner described an exceedingly rare anomaly found in his practice. A boy, age twelve, myopic from birth, with ectropion and total absence of the iris in both eyes. Both lenses were dislocated upwards, and for the last few months, cataractus. There was abundant room below each of the opaque lenses for the admission of light, and useful vision, which he did not have before, given him by the adjustment of proper glasses.

B. F. Church, M. D., Secretary.

